# Musculoskeletal Complaints Among Female Childcare Workers in German Daycare Centres—A Survey Study with a Comparison Group

**DOI:** 10.3390/ijerph22020270

**Published:** 2025-02-12

**Authors:** Roxana Dauer, Anja Schablon, Albert Nienhaus

**Affiliations:** 1 Competence Centre for Epidemiology and Health Services Research for Healthcare Professionals (CVcare), Institute for Health Services Research in Dermatology and Nursing (IVDP), University Medical Centre Hamburg-Eppendorf (UKE), 20246 Hamburg, Germany; a.schablon@uke.de (A.S.); a.nienhaus@uke.de (A.N.); 2 Department of Occupational Medicine, Hazardous Substances and Public Health (AGG), Institution for Statutory Accident Insurance and Prevention in the Health and Welfare Services (BGW), 22089 Hamburg, Germany

**Keywords:** cross-sectional survey, childcare workers, musculoskeletal diseases, occupational health, women’s health

## Abstract

Childcare workers are at risk of musculoskeletal complaints due to various stresses. Comparative studies focusing on this group are lacking. In a cross-sectional study, we asked female childcare workers and women from the general population (comparison group) about musculoskeletal complaints, categorised by body regions, and private/occupational risk factors. The childcare workers were recruited from Hamburg daycare centres and the comparison group from the residents’ registration office. The survey was conducted between October 2022 and July 2023. Odds ratios (ORs) were calculated in logistic regressions for factors influencing the occurrence of complaints that limited participants’ work or leisure activities in the past 12 months. Questionnaires from 218 female childcare workers and 250 from the comparison group were analysed. The comparison group had a 17.3% response rate. No response rate could be calculated for childcare workers. ORs for childcare workers were statistically significantly higher for complaints relating to the neck/cervical spine, shoulders/upper arms, knees and lumbar spine/lower back (OR between 1.7 and 3.2). This is the first study to compare the prevalence of musculoskeletal complaints in female childcare workers with other working women. The results show statistically significant differences, highlighting the need for interventions that address individual and workplace factors.

## 1. Introduction

Childcare workers in daycare centres face ergonomically unfavourable conditions and physical strain [1,2,3,4,5,6,7,8,9,10,11,12]. They sit on low chairs designed for children, often squat down to be at eye level with the children, interact with them on the floor and regularly lift and carry them. These workplace-specific stresses can lead to physical complaints that have been documented in the literature [13,14,15,16,17,18]. In a survey of childcare workers (*n* = 269) in daycare centres, Sinn-Behrend et al. [5] found that three-quarters of the respondents suffered from musculoskeletal health problems in at least one region of the body. A modified version of the Nordic Musculoskeletal Questionnaire was used in the survey, which asked about the frequencies of past complaints in different parts of the body. The authors emphasise that 51.6% of the respondents indicated at least one part of the back as a body region where they had experienced complaints. In addition, the frequency of complaints in the knee joint area was notable, with a total (unilateral and bilateral) of over 49%. This was significantly higher than in occupations involving heavy knee strain, such as those in the construction sector, where 33% of respondents reported complaints. Indications that childcare workers’ knee joints are exposed to particular stresses also emerged in the computer-aided job analysis carried out in the Sinn-Behrend study. On average, childcare workers spent 16% of a working shift in a kneeling position. Laudanski et al. [1], who used video observations to investigate the squatting behaviour of childcare workers, came to similar conclusions. The childcare workers (*n* = 18) spent more than a third of the observation period (3.25 h per participant) in a weight-bearing squatting posture. The authors point out that repetitive stress on the knee joints has been identified as risky in terms of developing micro-injuries in the knee. In the long term, these micro-injuries could lead to degenerative knee joint diseases and the development of osteoarthritis.

In addition, studies suggest that the psychological distress of childcare workers is also high and that there is a link between physical complaints and psychological distress [4,19].

Despite these findings, and to the best of our knowledge, there is still a lack of studies that specifically include other occupational groups in their study design and directly compare work-related complaints. Our study fills this gap by explicitly investigating the incidence and risk of musculoskeletal complaints in female childcare workers compared with a control group from the general female population. The present study examines the following questions:

- How frequently do female childcare workers in daycare centres report musculoskeletal complaints, particularly in the knee joint, compared to a female comparison group from the general population?
- To what extent does the job of a female childcare worker in daycare centres increase the risk of musculoskeletal complaints, especially in the knee joint, compared to a female comparison group from the general population?

## 2. Methods

### 2.1. Data Collection

From October 2022 to July 2023, we conducted a cross-sectional questionnaire survey of female childcare workers in daycare centres and women from other professional groups in the general population. Participants were able to take part both online and on paper. The study was an anonymous survey. It was not possible to draw any conclusions about the participants based on the data provided. The target group comprised female educational professionals aged between 40 and 67 and employed in a daycare centre setting. The study deliberately focused on women, as, on average, in 2022, 92.1% of the educational staff employed in daycare centres in Germany were women [20]. Women of the same age from other occupational groups in the general population were recruited as a comparison group. We set the age range at 40 to 67 years, as work-related musculoskeletal complaints tend to occur more frequently at an advanced age. The upper age limit of 67 years was chosen as this corresponds to the statutory retirement age and the study has an occupational health focus. This is to ensure that the results are relevant to the working population. Initially, participants were to be recruited from Hamburg and Magdeburg, but due to overlapping other occupational health surveys in Magdeburg, we focused on Hamburg. The City of Hamburg’s website was used to identify 1156 daycare centres and their contact details, from which a random sample of 446 was selected. After invitations to participate in the study were sent to the daycare centres, the respective daycare centre managers were contacted by telephone. We recruited the comparison group from the general population via the Hamburg residents’ registration office. For this purpose, 1500 women aged between 40 and 67, randomly selected from the population register based on the German Federal Registration Act, were contacted by post. The address data were used exclusively for sending invitations to participate in the study and were deleted after the study documents had been sent.

### 2.2. Dependent and Independent Variables

To document musculoskeletal complaints, the German version of the Nordic Musculoskeletal Questionnaire [21] was used to assess the cervical spine and neck, shoulder joints and upper arms, thoracic spine, lumbar spine and lower back, hip joints and thighs, and knee joints. Demographic and lifestyle characteristics such as gender, age, body mass index (BMI), number of children in the household, duration of weekly exercise, smoking status, vocational training, and job satisfaction were surveyed as potential confounders. Gender was defined by self-identification only. We did not collect data on sex assigned at birth to respect the diversity of gender identities. Participants had the option to indicate female, male, or diverse as their gender identity. Job satisfaction was measured using the associated COPSOQ scale and included aspects such as career prospects, colleagues, physical working conditions, department management, utilisation of personal skills, salary, and an overall assessment [22]. The dependent variable (outcome) used in the correlation analyses was discomfort in the various regions of the body that had led to restrictions in the participants’ professional activities or leisure time in the last 12 months.

The data collection was anonymised and is therefore not subject to the guidelines of the General Data Protection Regulation (GDPR) on the processing of personal data (Art. 4 and Recital 26 GDPR). As this was a survey study, no ethics application to a medical association was required and the Declaration of Helsinki was complied with. The data were collected and analysed anonymously. The data privacy regulations were adhered to.

### 2.3. Statistical Methods

The statistical evaluation of the collected data was carried out using SPSS version 29.0.1.0 software and included both descriptive (*t*-test for unconnected random samples) and multivariate analyses (logistic regressions), which were used to calculate odds ratios (OR) and 95% confidence intervals (CI). A significance level of *p* < 0.05 was also defined for the other statistical analyses.

## 3. Results

### 3.1. Study Population

Questionnaires from 218 female childcare workers from daycare centres and 250 women from other occupational groups, as a comparison group, were included in the data analysis. No participant identified as diverse. The response rate in the comparison group was 17.3%. As the number of childcare workers in the respective daycare centres was not known, no response rate could be calculated for this group. The average age of the childcare workers was 52 years (SD 7.5) and was statistically significantly lower than in the comparison group (54 years; SD 7.8; independent *t*-test, two-sided *p*-value < 0.01). The average body mass index (BMI) of the female childcare workers was 26.3 (SD 5.8), while that of the comparison group was 25.5 (SD 5.1). An independent *t*-test showed no significant difference between the groups in the comparison of averages (two-sided *p*-value = 0.112). The median value for both groups was just above the threshold value for “normal weight”. The group with a normal weight is the largest, with 51.4% of the childcare workers and 50.8% of the comparison group (Table 1).

In both groups, the majority of the childcare workers (51.4%) and the comparison group (60%) state that there are no children living in their household. There are no statistically significant differences between both of the groups. More childcare workers do no exercise (17%) or less than one hour per week (17%) compared to the participants in the comparison group (15% in each case). In addition, a higher proportion (27%) of the comparison group engaged in more intensive sporting activities for two to four hours per week than the childcare workers (16%). There are significant differences between the two groups (*p* = 0.034), which indicates that childcare workers are less physically active overall (Table 1). People who had never smoked made up the largest proportion in both groups (childcare workers: 48%, comparison group: 48%); 22% of the childcare workers smoke daily or occasionally, compared to 17% of the comparison group. In addition, fewer childcare workers are ex-smokers (30%) compared to the comparison group (34%). There are no statistically significant differences between the two groups (Table 1). In both groups, the proportion of participants who are currently in vocational training or do not have a vocational qualification is low, at less than 5% in each group. A significantly higher proportion of childcare workers have completed vocational training (76%) compared to the comparison group (52%). The differences in the distribution of professional qualifications between the comparison group and the childcare workers are statistically significant (*p* < 0.001) (Table 1). Among the educational staff in the daycare centres, 75% state that they are educators, 6% are child carers, 5% are socio-educational assistants, 4% are remedial therapists, 4% are social education workers, and 1% are social assistants. In the comparison group, the three most frequently cited sectors are medical healthcare professions (14%), professions in financial services, accounting and tax consultancy (11%), and professions in law and administration (9%).

### 3.2. Results Regarding Musculoskeletal Complaints

**Figure 1:**
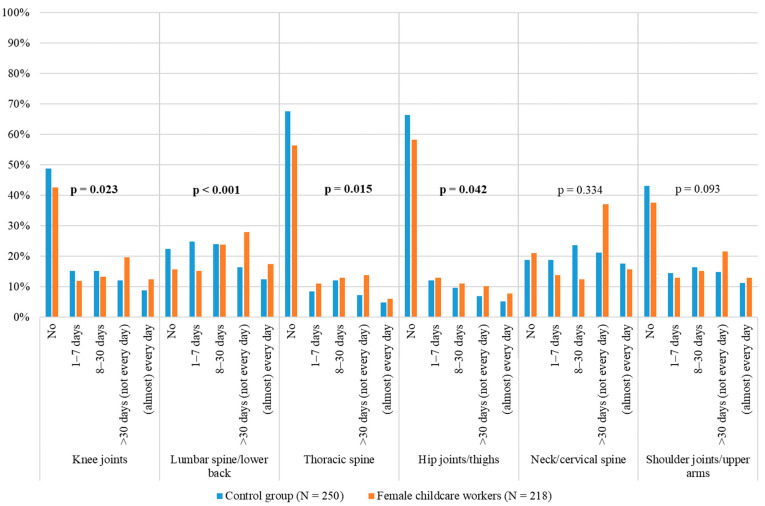
Group comparison (childcare workers/comparison group): information on days when complaints causing restrictions at work and during leisure time were experienced in the previous 12 months, per body region.

The evaluation of the Nordic Musculoskeletal Questionnaire showed that there are differences in the prevalence of musculoskeletal complaints between both groups. Figure 1 shows the number of days on which complaints have been experienced in the previous 12 months, per body region. Across all body regions, the childcare workers selected the categories “more than 30 days” and “(almost) every day” more frequently than the participants in the comparison group.

There are statistically significant differences between the two groups with regard to the knee joints, the lumbar spine and lower back, the thoracic spine, and the hip joints and thighs. With the exception of the neck and cervical spine region, the participants in the comparison group reported more frequently than the childcare workers that they had no discomfort in the other body regions. With regard to knee joint discomfort, 20% of the childcare workers stated that they had experienced problems on “more than 30 days” in the past year, compared with 12% in the comparison group. Furthermore, 12% of the childcare workers report experiencing discomfort “(almost) every day”, while this is the case for 9% of the comparison group.

The evaluation of occupational or leisure time restrictions due to complaints in the last year by body region, as shown in Table 2, reveals that the childcare workers are statistically significantly more likely than the comparison group to state that they are affected by restrictions in everyday life in all the body regions examined. In the knee area, 39% of the childcare workers reported restrictions, compared with 27% of the comparison group.

In the regression analyses, being in the childcare workers’ occupational group had a statistically significant influence on complaints in almost all the body regions examined (Table 3 and Table 4). Exceptions are the thoracic spine and the hip joints and thighs, for which we found no statistically significant results with regard to occupation as an influencing factor.

The OR for experiencing restrictions due to knee pain in everyday life is 1.8 (95% CI 1.19–2.81) if the person works as a childcare worker in a daycare centre (Table 4). Another statistically significant risk factor for knee joint restrictions is a BMI in the range of obesity grade I–II. The highest OR is found in grade II (OR 4.6; 95% CI 1.63–13.00). The prevalence of restrictions due to knee complaints also increases with advancing age. For the 50 to 59 age group, the OR is 2.0 (95% CI 1.15–3.38) and this rises to 2.8 (95% CI 1.47–5.45) for the 60 to 67 age group. Participants who exercise regularly reported more frequent restrictions due to knee pain in everyday life than those who do not exercise. However, the OR is only statistically significant in the group that exercises for less than one hour per week (OR 2.2; 95% CI 1.04–4.67). The OR for restrictions due to knee complaints in everyday life is 2.2 (95% CI 1.20–3.89) if a child is living in the household and is statistically significant. Additional children do not increase the OR. There were no statistically significant correlations with job satisfaction for restrictions due to knee complaints in everyday life.

For other body regions, the potential confounders described above—BMI, age, weekly physical activity, and children in the household—only occasionally showed statistically significant influences on complaints. For thoracic spine complaints, the OR is reduced for the 60 to 67 age category (OR 0.3; 95% CI 0.12–0.58). In the lumbar spine and lower back, grade I obesity had an increased effect (OR 2.0; 95% CI 1.05–3.73). In addition, the OR for the 50 to 59 age category is increased for discomfort in the hip joints and thighs (OR 2.0; 95% CI 1.07–3.60).

For the confounder job satisfaction, statistically significant correlations were found for all body regions examined, with the exception of the knee joints. People with a very high level of job satisfaction were less likely to report discomfort in the neck and cervical spine (OR 0.2; 95% CI 0.09–0.31), shoulder joints and upper arms (OR 0.4; 95% CI 0.20–0.65), thoracic spine (OR 0.4; 95% CI 0.22–0.89), lumbar spine and lower back (OR 0.4; 95% CI 0.23–0.74), and hip joints and thighs (OR 0.2; 95% CI 0.08–0.50), compared with people who reported very low job satisfaction.

## 4. Discussion

This is the first study to compare the prevalence of musculoskeletal complaints in female childcare workers with that of other working women. The results show statistically significant differences in the frequency of musculoskeletal complaints between the two groups. Female childcare workers in particular frequently report longer and more persistent complaints in the knee joints, lumbar spine and lower back, thoracic spine, and hip joints and thighs. This suggests that the professional demands of female childcare workers are potentially detrimental to their health. The regression analyses showed that after controlling for potential confounders, working as a childcare worker is a statistically significant risk factor for musculoskeletal complaints in all body regions examined, with the exception of the thoracic spine and hip joints and thighs. Our results confirm previous studies that have found a high prevalence of musculoskeletal complaints among educational staff [1,2,3,4,5,6,8,9,10,13,14,15,17,19]. Particularly noteworthy is the great strain on the knee joints, the lumbar spine and the lower back, typically caused by frequently lifting and carrying children, as well as long periods of standing and bending down in daycare centres. This problem is discussed in the literature and there are indications that ergonomic interventions and preventive measures could provide a remedy in this case [1,2,5,11,12,24]. However, it is striking that almost all parts of the bodies of female childcare workers are affected. This suggests that there are other factors that affect musculoskeletal discomfort in general, without having a specific effect on individual regions. Mental stress at work or an imbalance between stress and reward should be discussed [10,19]. Our evaluations with regard to job satisfaction indicate that there is a correlation here. Our results suggest that high job satisfaction tends to be associated with a lower risk of discomfort and may have a protective effect on the occurrence of musculoskeletal complaints.

The professional framework conditions for childcare workers in daycare centres indicate a high workload. An analysis of sick leave reports in Germany for 2023 has shown that childcare centre workers are absent due to illness significantly more often than all other occupational groups, with an average of around 30 days a year compared to an average of around 20 days for the other groups [25]. There are also around 440,000 unfilled daycare vacancies across Germany [26] and a severe shortage of skilled personnel. It can be assumed that, in view of these working conditions, the pressure on those daycare centre workers who actively pursue this profession is high and increases their workload, which can be a risk factor for physical complaints. The high prevalence of musculoskeletal complaints among childcare workers has implications for work design and health promotion in daycare centres. Our study highlights the need for preventive measures to reduce the physical strain on female childcare workers.

### Strengths and Weaknesses of the Study

One of the strengths of this study is that, for the first time, a comparison group was used to investigate the prevalence and severity of musculoskeletal complaints among female childcare workers in daycare centres. The comparison with a general population group made it possible to estimate the risk of musculoskeletal complaints associated with working as a childcare worker, confirming the need for preventive measures. Additionally, there is limited research on workplace stress in predominantly female occupations. Our study helps to close this gap by specifically analysing the working conditions and health challenges in an occupational field dominated by women and by deliberately focusing on women. More research is needed in this area to raise awareness of gender-specific differences in workload and its impact on health and to create a basis for developing preventive and health-promoting measures. This could also help to promote gender equality in the workplace.

One limitation of our study is the potential selection bias. As participation in the study was voluntary, people who already had medical complaints may have been more inclined to take part in the survey. However, this tendency is likely to apply to both the childcare workers and the participants in the comparison group. This could possibly mean that the prevalence of musculoskeletal complaints in both groups is overestimated. In addition, the data collected is based on participants’ self-assessments. This can be influenced by individual differences in perception. Another limitation arises from the control group composition. It included participants from very different sectors, each with diverging working conditions, some of which also put strain on the musculoskeletal system. As a result, possible differences between childcare workers and the participants in the comparison group tend to be underestimated. As this is a cross-sectional study, causal interpretations can only be made with caution.

## 5. Conclusions

Our study indicates that childcare workers are at increased risk of musculoskeletal complaints. More research is needed to identify typical straining tasks. That said, our data call for measures to reduce the complaints of childcare workers, targeting both individual factors and strategies to improve the working conditions. Future research should focus on developing and evaluating such interventions, taking into account long-term effects on the health and ability to work of female childcare workers.

## Figures and Tables

**Table 1:** Descriptive statistics on the study population.

Variable		Comparison Group *n* = 250 (%)	Childcare Workers *n* = 218 (%)	*p*-Value ^1^
Age	40 to 49 years	75 (30.0)	82 (37.6)	**0.007**
	50 to 59 years	94 (37.6)	93 (42.7)	
	60 to 67 years	81 (32.4)	43 (19.7)	
BMI ^2^	Underweight (<18.5)	8 (3.2)	2 (0.9)	0.258
	Normal weight (18.5–24.9)	127 (50.8)	112 (51.4)	
	Overweight (25–29.9)	69 (27.6)	56 (25.7)	
	Grade I obesity (30–34.9)	30 (12.0)	33 (15.1)	
	Grade II obesity (35–39.9)	12 (4.8)	7 (3.2)	
	Grade III obesity (>40)	4 (1.6)	8 (3.7)	
Children in the household	No children	150 (60.0)	112 (51.4)	0.228
	1 child	41 (16.4)	43 (19.7)	
	2 children	48 (19.2)	47 (21.6)	
	3+ children	11 (4.4)	16 (7.3)	
Sports/exercise: weekly activity	No sporting activity	38 (15.2)	37 (17.0)	**0.034**
	<1 h	38 (15.2)	37 (17.0)	
	1–2 h	66 (26.4)	76 (34.9)	
	2–4 h	68 (27.2)	34 (15.6)	
	>4 h	40 (16.0)	34 (15.6)	
Smoking	Yes, daily, or occasionally	43 (17.2)	47 (21.6)	0.494
	Ex-smoker	86 (34.4)	65 (29.8)	
	Never	120 (48.0)	104 (47.7)	
	Not specified	1 (0.4)	2 (0.9)	
Educational qualification	Basic education ^3^	2 (0.8)	1 (0.5)	0.052
	Secondary school certificate ^4^	17 (6.8)	8 (3.7)	
	Middle maturity	67 (26.8)	82 (37.6)	
	Qualification to enter higher education	159 (63.6)	119 (54.6)	
	Other educational qualification	5 (2.0)	8 (3.7)	
Vocational training and instruction	Currently in vocational training ^5^	1 (0.4)	1 (0.5)	**<0.001**
	No vocational qualification	5 (2.0)	1 (0.5)	
	Vocational training and instruction ^6^	130 (52.0)	166 (76.1)	
	Higher education ^7^	103 (41.2)	43 (19.7)	
	Other vocational qualification	11 (4.4)	7 (3.2)	
Job satisfaction	Very low	57 (22.8)	56 (25.7)	**0.009**
	Low	55 (22.0)	66 (30.3)	
	High	65 (26.0)	60 (27.5)	
	Very high	73 (29.2)	36 (16.5)	

^1^ Pearson’s chi-square test; *p*-values that are statistically significant have been highlighted in bold. ^2^ Limit values of the German Obesity Society (DAG) [23]. ^3^ No educational certificate. ^4^ German: Hauptschulabschluss. ^5^ Apprentices, students, vocational preparation year. ^6^ Vocational/in-company training, vocational/school training, training at a technical college/master school. ^7^ Bachelor’s degree from a university (of applied sciences), another degree from a university of applied sciences, another degree from a university/college.

**Table 2:** Occupational or leisure time restrictions due to complaints in the last year by body region.

Variable		Comparison Group *n* = 250 (%)	Childcare Workers *n* = 218 (%)	*p*-Value ^1^
Neck/cervical spine	Yes	95 (38.0)	125 (57.3)	**<0.001**
	No	155 (62.0)	93 (42.7)	
Shoulder joints/upper arms	Yes	73 (29.2)	92 (42.2)	**0.003**
	No	177 (70.8)	126 (57.8)	
Thoracic spine	Yes	46 (18.4)	58 (26.6)	**0.033**
	No	204 (81.6)	160 (73.4)	
Knee joints	Yes	67 (26.8)	85 (39.0)	**0.005**
	No	183 (73.2)	133 (61.0)	
Lumbar spine/lower back	Yes	99 (39.6)	147 (67.4)	**<0.001**
	No	151 (60.4)	71 (32.6)	
Hip joints/thighs	Yes	38 (15.2)	55 (25.2)	**0.007**
	No	212 (84.8)	163 (74.8)	

^1^ *p*-values that are statistically significant have been highlighted in bold.

**Table 3:** Logistic regressions on occupational or leisure time restrictions due to complaints (yes/no) in the last year by body region (*n* = 468)—upper extremities.

Variable		Neck/Cervical Spine		Shoulder Joints/Upper Arms		Thoracic Spine	
		OR (95% CI)	*p*-Value ^1^	OR (95% CI)	*p*-Value ^1^	OR (95% CI)	*p*-Value ^1^
Group	Comparison group (ref.) ^2^/ childcare worker	**1.9 (1.27–2.85)**	**0.002**	**1.7 (1.12–2.52)**	**0.012**	1.3 (0.84–2.15)	0.225
BMI	Normal weight (ref.) ^2^	–	0.637	–	0.813	–	0.711
	Underweight	0.3 (0.06–1.59)	0.156	0.6 (0.11–2.93)	0.494	0.8 (0.15–4.04)	0.771
	Overweight	1.2 (0.73–1.91)	0.495	1.3 (0.78–2.03)	0.352	1.2 (0.66–2.06)	0.588
	Grade I obesity	1.2 (0.65–2.24)	0.545	1.4 (0.75–2.50)	0.312	1.6 (0.82–3.22)	0.164
	Grade II obesity	0.8 (0.28–2.12)	0.605	0.9 (0.32–2.72)	0.907	1.5 (0.47–4.56)	0.506
	Grade III obesity	1.0 (0.27–3.43)	0.945	1.1 (0.30–3.70)	0.934	1.9 (0.55–6.91)	0.303
Age	40–49 years (ref.) ^2^	–	0.454	–	0.449	–	**0.002**
	50–59 years	1.3 (0.78–2.12)	0.328	1.4 (0.82–2.26)	0.226	0.8 (0.47–1.39)	0.440
	60–67	1.0 (0.53–1.79)	0.939	1.4 (0.74–2.55)	0.310	**0.3 (0.12–0.58)**	**<0.001**
Sport/week	No sport (ref.) ^2^	–	0.346	–	0.848	–	0.754
	<1 h	1.3 (0.63–2.50)	0.524	1.2 (0.58–2.31)	0.683	0.9 (0.38–1.99)	0.736
	1–2 h	1.5 (0.82–2.75)	0.184	1.1 (0.59–1.97)	0.816	1.2 (0.61–2.51)	0.562
	2–4 h	1.1 (0.58–2.16)	0.736	0.9 (0.46–1.79)	0.785	1.4 (0.64–3.00)	0.405
	>4 h	0.8 (0.40–1.64)	0.551	1.3 (0.65–2.68)	0.437	1.0 (0.43–2.34)	0.989
Children	No children in household (ref.) ^2^	–	0.281	–	0.902	–	0.780
	1 child in household	0.8 (0.45–1.41)	0.440	1.0 (0.55–1.72)	0.916	0.7 (0.38–1.40)	0.342
	2 children in household	0.9 (0.49–1.60)	0.684	1.1 (0.60–1.97)	0.772	0.8 (0.42–1.51)	0.480
	3+ children in household	2.1 (0.81–5.23)	0.131	1.4 (0.55–3.43)	0.495	0.8 (0.27–2.26)	0.656
Job satisfaction	Very low (ref.) ^2^	–	**<0.001**	–	**0.002**	–	**0.011**
	Low	**0.5 (0.29–0.89)**	**0.018**	**0.5 (0.29–0.87)**	**0.014**	0.9 (0.52–1.72)	0.855
	High	**0.3 (0.19–0.57)**	**<0.001**	**0.4 (0.25–0.75)**	**0.003**	**0.4 (0.22–0.83)**	**0.012**
	Very high	**0.2 (0.09–0.31)**	**<0.001**	**0.4 (0.20–0.65)**	**<0.001**	**0.4 (0.22–0.89)**	**0.022**

^1^ *p*-values that are statistically significant have been highlighted in bold. ^2^ Reference category for comparison with other categories in logistic regression analysis.

**Table 4:** Logistic regressions on occupational or leisure time restrictions due to complaints (yes/no) in the last year by body region (*n* = 468)—lower extremities.

Variable		Knee Joints		Lumbar Spine/Lower Back		Hip Joints/Thighs	
		OR (95% CI)	*p*-Value ^1^	OR (95% CI)	*p*-Value ^1^	OR (95% CI)	*p*-Value ^1^
Group	Comparison group (ref.) ^2^/ childcare worker	**1.8 (1.19–2.81)**	**0.006**	**3.2 (2.09–4.80)**	**<0.001**	1.6 (0.98–2.60)	0.062
BMI	Normal weight (ref.) ^2^	–	**0.002**	–	0.072	–	0.728
	Underweight	0.9 (0.18–4.89)	0.934	0.3 (0.05–1.71)	0.173	0.5 (0.06–4.22)	0.508
	Overweight	1.5 (0.89–2.45)	0.132	1.6 (0.95–2.53)	0.078	0.9 (0.47–1.56)	0.606
	Grade I obesity	**2.9 (1.59–5.46)**	**<0.001**	**2.0 (1.05–3.73)**	**0.034**	1.1 (0.51–2.18)	0.889
	Grade II obesity	**4.6 (1.63–13.00)**	**0.004**	2.3 (0.83–6.61)	0.107	2.0 (0.66–5.81)	0.224
	Grade III obesity	3.3 (0.91–11.82)	0.070	1.1 (0.31–3.87)	0.880	1.5 (0.38–5.54)	0.585
Age	40–49 years (ref.) ^2^	–	**0.006**	–	0.499	–	0.053
	50–59 years	**2.0 (1.15–3.38)**	**0.013**	1.3 (0.81–2.25)	0.255	**2.0 (1.07–3.60)**	**0.029**
	60–67	**2.8 (1.47–5.46)**	**0.002**	1.3 (0.72–2.49)	0.357	1.2 (0.56–2.50)	0.657
Sport/week	No sport (ref.) ^2^	–	0.317	–	0.393	–	0.375
	<1 h	**2.2 (1.04–4.67)**	**0.040**	1.5 (0.75–3.14)	0.239	1.2 (0.51–2.62)	0.739
	1–2 h	1.8 (0.92–3.55)	0.086	1.2 (0.63–2.14)	0.631	1.3 (0.63–2.59)	0.506
	2–4 h	1.6 (0.79–3.41)	0.186	0.9 (0.45–1.69)	0.682	0.6 (0.27–1.48)	0.291
	>4 h	1.9 (0.86–4.15)	0.113	1.5 (0.73–3.05)	0.273	0.8 (0.32–1.91)	0.594
Children	No children in household (ref.) ^2^	–	0.076	–	0.390	–	0.956
	1 child in household	**2.2 (1.20–3.89)**	**0.010**	0.7 (0.38–1.22)	0.202	0.9 (0.46–1.78)	0.778
	2 children in household	1.2 (0.62–2.20)	0.639	0.9 (0.49–1.66)	0.741	0.8 (0.41–1.71)	0.627
	3+ children in household	1.3 (0.47–3.52)	0.624	0.5 (0.20–1.32)	0.169	0.8 (0.24–2.67)	0.709
Job satisfaction	Very low (ref.) ^2^	–	0.257	–	**<0.001**	–	**0.005**
	Low	0.7 (0.39–1.23)	0.208	1.1 (0.64–2.02)	0.665	0.8 (0.42–1.47)	0.443
	High	0.7 (0.38–1.19)	0.173	**0.4 (0.25–0.78)**	**0.004**	0.9 (0.49–1.67)	0.753
	Very high	0.5 (0.30–1.01)	0.054	**0.4 (0.23–0.74)**	**0.003**	**0.2 (0.08–0.50)**	**<0.001**

^1^ *p*-values that are statistically significant have been highlighted in bold. ^2^ Reference category for comparison with other categories in logistic regression analysis.

## Data Availability

The datasets presented in this article are not available due to privacy restrictions. Participants have been assured that their information will not be disclosed to third parties. Requests should be addressed to the corresponding author.

## Abbreviations

BMI: body mass index; OR: odds ratio; SD: standard deviation; 95% CI: 95% confidence interval.
